# Decreased osteoblastogenesis from synovial fluid progenitors as a marker of systemic inflammatory process in juvenile idiopathic arthritis

**DOI:** 10.1186/1546-0096-9-S1-P311

**Published:** 2011-09-14

**Authors:** E Lazic Mosler, M Jelusic-Drazic, D Grcevic, A Marusic, N Kovacic

**Affiliations:** 1 University of Zagreb School of Medicine, Zagreb, Croatia; 2 University Hospital Centre, Zagreb, Croatia; 3 University of Split School of Medicine, Split, Croatia

## Background

Juvenile idiopathic arthritis (JIA) is characterized by synovial hyperplastic changes, which may contribute to joint destruction by inhibiting osteoblastogenesis.

## Aims

The aims of this report are: 1) to assess osteoblastogenesis from synovial fluid (SF) progenitors in children with JIA, 2) to assess the effect of SF from patients with JIA on osteoblastogenesis from bone marrow (BM) progenitors, and 3) to assess local and systemic expression of OBL related genes in JIA.

## Methods

Peripheral blood (PB) samples were obtained from children with oligoarticular JIA (oJIA, n=18), polyarticular JIA (pJIA, n=20), and healthy controls (n=18). SF samples were collected from children with oJIA (n=20) and pJIA (n=7). Osteoblastogenesis was induced with 50 μg/ml ascorbic acid and 5 mmol β-glycerophosphate, in SF cells and BM cells obtained from a healthy donor, and assessed by alkaline phosphatase (AP) histochemistry. Gene expression of Runx-genes, osteoprotegerin (OPG) and receptor activator of nuclear factor-κB ligand (RANKL) was analyzed by qPCR.

## Results

Osteoblastogenesis from SF cells was higher in children with oJIA, than in pJIA (784.81±216.79 vs. 257.21±68.13 units, p<0.001, t-test), and negatively correlated with erythrocyte sedimentation rate (ρ=–0.4139, p=0.03). SF from children with oJIA and pJIA inhibited osteoblastogenesis from bone marrow (0.059±0.026 in oJIA; 0.068±0.019 in pJIA vs. 0,115 ± 0,023 in untreated cultures, p<0.05, t-test). Expression of Runx1 and RANKL was higher in SF cells from pJIA than from oJIA patients (p<0.05, Mann-Whitney test).

## Conclusion

Osteoblast differentiation is locally inhibited in JIA, particularly in children with pJIA, and correlate with systemic inflammatory activity.

